# In-operando high-speed microscopy and thermometry of reaction propagation and sintering in a nanocomposite

**DOI:** 10.1038/s41467-019-10843-4

**Published:** 2019-07-10

**Authors:** Haiyang Wang, Dylan J. Kline, Michael R. Zachariah

**Affiliations:** 1 Department of Chemical and Environmental Engineering, University of California, Riverside, CA 92521 USA; 2 Department of Chemical and Biomolecular Engineering, University of Maryland, College Park, MD 20742 USA

**Keywords:** Nanoparticles, Metals and alloys, Imaging techniques

## Abstract

An important proposed mechanism in nanothermites reactions — reactive sintering — plays a significant role on the combustion performance of nanothermites by rapidly melting and coalescing aggregated metal nanoparticles, which increases the initial size of the reacting composite powders before burning. Here, we demonstrate a high-speed microscopy/thermometry capability that enables ~ µs time and ~ µm spatial resolution as applied to highly exothermic reaction propagation to directly observe reactive sintering and the reaction front at high spatial and temporal resolution. Experiments on the Al+CuO nanocomposite system reveal a reaction front thickness of ~30 μm and temperatures in excess of 3000 K, resulting in a thermal gradient in excess of 10^7^ K m^−1^. The local microscopic reactive sintering velocity is found to be an order of magnitude higher than macroscale flame velocity. In this observed mechanism, propagation is very similar to the general concept of laminar gas reaction theory in which reaction front velocity ~ (thermal diffusivity x reaction rate)^1/2^.

## Abstract (web summary)

Imaging highly exothermic reactions in high spatial and temporal resolution to understand their underlying reaction mechanisms is challenging. Here, the authors develop a high-speed microscopy/pyrometry imaging system to successfully observe reactive sintering in a nanothermite reaction in-operando.

## Introduction

Compared to conventional explosives containing carbon, hydrogen, nitrogen, and oxygen, such as TNT, RDX, and HMX, energetic nanocomposites such as nanothermites are attracting more attention for their high enthalpy of reaction and environmentally benign products^1–7^. Their high surface area gives them a substantially higher energy release rate in comparison to their micron counterparts, thus making them potential candidates for materials whose reactivity fall in between primary explosives and conventional pyrotechnics. This unique category, which has both military and civilian applications, is limited in widespread implementation due to the inherent complexity of heterogeneous combustion that has yet to be fully understood. For example, theoretically, when reducing the size of the composition constituents to the nanoscale, the energy release rate should be enhanced by orders of magnitudes due to a highly increased interfacial area and reduced diffusion distance between fuel and oxidizer^2,8,9^; however, they have yet to exhibit such impressive enhancements^10–12^. One of the major concerns that may explain this underwhelming enhancement is the loss of nanostructure during reaction.

Sullivan et al.^13^ found that an important component in the combustion of nanothermites — reactive sintering — plays a significant role by rapidly melting and coalescing aggregated aluminum nanoparticles and increases the initial size of the reacting composite powders before burning (sintering time ≤ reaction time). Further studies by Chakraborty and Zachariah^14^ conducted a reactive molecular dynamics simulation and found that the sintering behavior was motivated by an induced built-in electric field. The loss of nanostructure due to reactive sintering in nanothermite powders dramatically impacts the combustion of the composite and, as a result, burn times of the materials do not significantly shorten as a function of diameter and small particle sizes^10–12^. Other studies have shown that the fractional scaling law observed for the gas-phase burning of nanoparticles can be corrected for by considering sintering effects prior to burning^15^. Egan et al.^16^ used dynamic transmission electron microscopy (TEM) to observe morphological changes occurring in nanoparticle aggregates and found that the coarsening process initiates within 15 ns and is completed in <50 ns; however, the heating in this case was by a laser and thermometry was unavailable. Even though the reactive sintering phenomenon was speculated in a motionless heating stage for TEM and was simulated by the related models, the direct observation of a dynamic reactive sintering process and subsequent propagation process in a nanothermite has not yet been observed in realistic operating conditions.

Numerous studies have been conducted with various approaches to directly observe the reaction dynamics of the nanothermites, but none have been able to practically probe a propagating reaction front or reactive sintering on a time or length scale commensurate with the phenomena. Some macroscale studies of thermite reaction dynamics have been captured by T-Jump/time-of-flight mass spectrometer developed by Zhou et al.^17^, which rapidly heats (~10^5^ K s^−1^) a material and can provide time-resolved ignition and reaction product profiles. Another commonly employed device to quantify reaction dynamics in bulk materials has been a specially modified constant volume combustion cell developed by Sullivan et al.^18^ to simultaneously measure the pressure and optical emission histories of a nanothermite reaction. However, while these measurements provide useful insight on the energy release rate and reaction mechanisms in materials on the appropriate reaction time scales (~µs), they were unable to capture the dynamics and observe the reaction at the particle length scale (µm/nm). Attempts to directly observe the reaction at these length scales have been performed using fast-heating stages for scanning electron microscopy (SEM) or TEM, which did show sintering before and after the heating; however, they were unable to resolve the event on a nanothermite reaction time scale (~µs)^16,19,20^. To our knowledge, a fast-response, in-operando technique has yet to be employed to observe nanothermite reactions at resolutions high enough to resolve particle-sized phenomena and on the reaction time scale (~µs).

In this paper, we introduce a technique that bridges the gap between in-operando fast response (µs) and high resolution (µm). In this technique, a microscope objective coupled to a high-speed color video camera enables us to observe the in-operando microscale reaction and propagation of nanocomposite thermites. The high-speed video (~55 µs per frame) of flame propagation and reactive sintering were captured within a 512 µm × 512 µm zone of reacting material with a resolution of 1 pixel: 1 µm. Furthermore, the color video could, through appropriate calibration and using the RGB filter within the camera, enable two-dimensional (2D) pyrometry temporal temperature maps of the reaction zone. Having imaged the reaction, the exact zone and the corresponding sintered particles were found in a SEM and matched perfectly with the particles found in the microscope. By employing these techniques, we provide images to demonstrate reaction propagation in nanothermites and reactive sintering on the microscale in both space and time.

## Results

### Evaluating the reaction zone

**Fig. 1:**
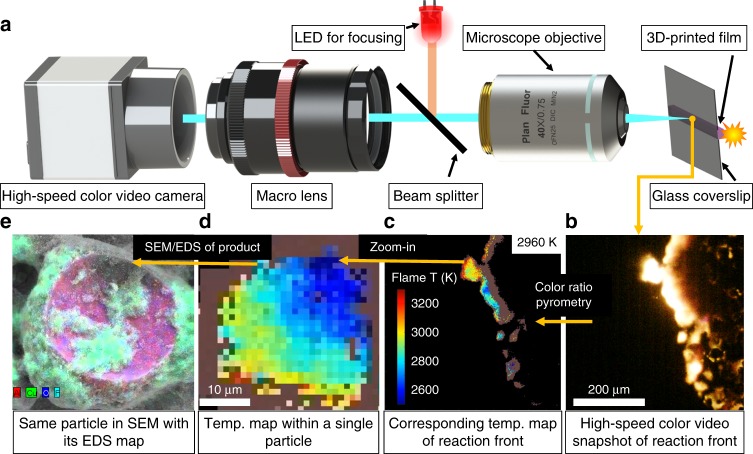
Imaging setup and example outputs. **a** Schematic showing high-speed microscope imaging of three-dimensionally (3D) printed reactive materials. Read from right to left: **b** High-speed color video snapshot of reaction front; **c** corresponding temperature map of reaction front; **d** temperature map within a single particle; and **e** the same particle in scanning electron microscopy (SEM) with its energy-dispersive spectrometry (EDS) map. CAD file of microscope objective courtesy of ThorLabs

Figure 1a shows the major configuration of this study, in which a key point is employing a ×40 microscope objective coupling with a high-speed video camera. The microscope objective was focused on the backside of a cover glass slide on which the sample film was printed, thereby allowing the visualization of the flame front without the generated products obscuring the view. With the microscope objective, the pixel/size ratio was ~1 µm/pixel. At this resolution, the flame front (Fig. 1b, c), as well as a single sintering particle (Fig. 1d, e), could be captured and the corresponding temperature map (Fig. 1c, d) could be obtained. The area was labeled by a thin marker before the ignition, thus allowing us to find the exact area later in a SEM (Fig. 1e). A summary of the kind of information is shown in the panel of images below: read from right to left, a snapshot taken from a single video frame (Fig. 1b), the corresponding temperature map (Fig. 1c) of the corresponding frame, temperature of single particle showing a thermal gradient within a particle (Fig. 1d), and finally the same particle imaged under SEM with its energy-dispersive spectrometry (EDS) map (Fig. 1e).

**Fig. 2:**
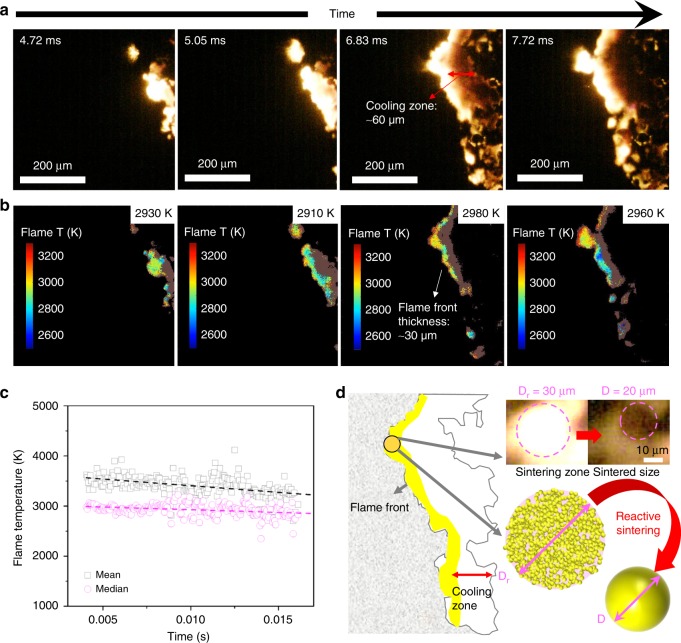
Images and temperature maps from high-speed microscopy video. **a** Series of snapshots of burning flame front. Labeled on the left-top corner is the time after flame front entering the zone (512 µm × 512 µm). **b** The corresponding flame temperature maps and the average temperature (in Kelvin). High error points were marked as brown and are excluded from the calculations. **c** The mean (square marker) and median (round marker) flame temperature with time (with reference lines). **d** Schematic showing reactive sintering and its spatiotemporal relationship to reaction propagation. Note: The propagation direction is from right to left

Figure 2a shows temporal reaction propagation snapshots of the Al/CuO nanothermites (90 wt%) captured in a zone of 512 µm × 512 µm with a frame rate of ~18,000 (55.5 µs per frame) and the corresponding flame temperature maps were obtained by a color camera pyrometry through image processing. Four typical frames and their temperature maps were selected and shown in Fig. 2a, b, respectively, from which we can see that the flame fronts consist of stochastic bright spots, which discontinuously propagate the reaction. These bright areas are roughly divided into a leading flame front and a following cooling zone, as distinguished by the brightness. The noticeably brighter area spanning ~30 µm was confirmed to be the leading edge from measured temperature ~3000 K, which is close to previously measured reaction temperature of Al/CuO^21^. As shown in Fig. 2c, the time-resolved temperature was found to be constant at ~3000 (median) and ~3500 K (mean) irrespective of flame front location, thus confirming that the reaction is relatively homogenous with a stable energy release despite the discontinuous front.

Reactive sintering, as discussed above and previously observed^13–16^, is the coalescence of aggregated and/or agglomerated nanoparticles driven by heat release during the reaction and results in the effective loss of nanostructure that was originally introduced to enhance reactivity. As shown in Fig. 2d, after the flame front has passed any given area, sintered particles with a mean diameter of ~20 µm are observed, representing a three order of magnitude increase in particle size from the nano-sized precursors. It is also notable that the flame front thickness is roughly the same size as the sintering zone (as defined by a noticeably bright/hot area where reactive sintering is occurring), a reasonable observation considering that these flame fronts were constructed by networks of individually sintering particles propagating the reaction by either advection or heat release. As schematically shown in Fig. 2d, the fact that the cooling zone is ~2–3 times larger than the flame front thickness (=sintering zone) indicates that the cooling time would be longer than the sintering time.

In Fig. 2d, the area of the bright flame ball is the sintering zone, which is effectively the flame front thickness. If we now consider the aggregate projected area (with a diameter of *D*_r_) as the sintering zone and the cooled particle (*D*) as the final sintered size, we can explore the relationship between *D*_r_ and *D*. For these materials (see Supplementary Fig. 1 for SEM image), we obtain a measured density of 33% theoretical density, which is consistent with our prior work^22–24^. At an aggregate packing density of 33%, coalescence will result in rough factor of 3 decrease in volume of the resulting sintered particle and 0.69 factor decrease in diameter, that is, *D* ~0.69*D*_r_. This result is remarkably consistent with the experimental results shown in the microscope image in Fig. 2d that the final sintered size (~20 µm) and the sintering zone is ~30 µm. The sintering zone is thus, for a thermite, the effective thickness of the reaction zone and represents the local volume of material reacting as an entity, resulting in a single sintered particle product.

### Mapping optical to electron microscopy

**Fig. 3:**
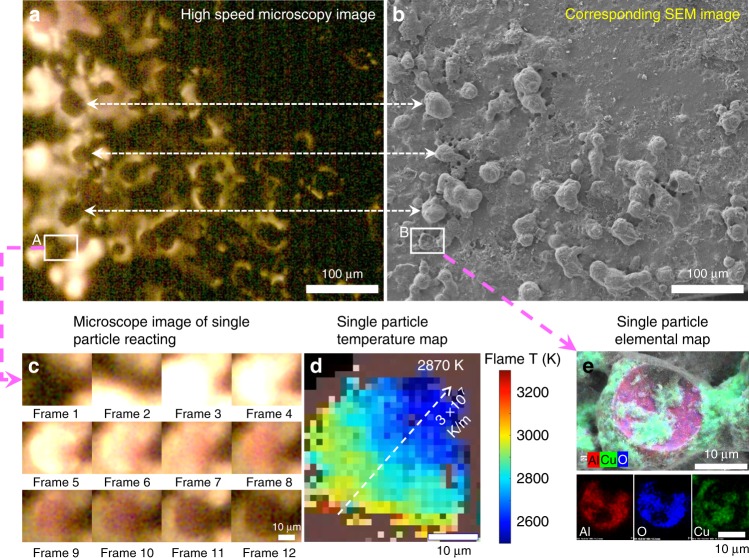
Optical microscope image and corresponding post-reaction scanning electron microscopy/energy-dispersive spectrometry (SEM/EDS) image with products. **a** Typical in-operando microscope image of Al/CuO reactive sintering, and after, **b** the corresponding SEM image of sintered combustion residue was found. **c** Series of snapshots of a single particle (55.55 µs per frame) showing the appearance of a large particle indicative of reactive sintering. **d** Intra-particle temperature at peak temperature showing a thermal gradient within the particle. **e** The EDS mapping of the above in-operando sintered Al_2_O_3_ particle coated with Cu nanoparticles, as evident by EDS mapping (red: Al; blue: O; green: Cu). Note: combustion is from right to left

To investigate the morphology, composition, and size distribution of the post-combustion product, the reaction product-coated slide was examined by SEM and EDS. As shown in Fig. 3a, b, we were able to find an exact one-for-one correspondence between the in-operando microscope imaging and the SEM image. This is an important result because it implies that we can conduct post-reaction forensics for composition, corresponding to the exact reaction conditions observed in the in-operando microscope imaging. The average size of these spherical particles is ~25 µm, confirmed by both optical and electron microscope. To see them more clearly, a typical particle was selected and marked as areas A and B in Fig. 3a, b, respectively, which will be discussed in detail later.

Area A in Fig. 3a was enlarged and the series of snapshots (55.5 µs per frame) are shown in Fig. 3c. To our knowledge, this series of images is the first observation of the temporal and spatial propagation of a thermite reaction at the microscale. Although the spatial resolution of this optical microscope is clearly inferior to an electron microscope, the image sequences are especially useful in that the whole reactive sintering process can be visually observed for the nanocomposite thermite, and from this, the sintering time, cooling time, and sintered size can be obtained. If we define the reactive sintering time as that from ignition to the time of brightest emission, the entire sintering process takes ~170 µs (3 frames) and the subsequent cooling time required an additional ~350 µs. The bright part in the images is much larger than the final sintered particle, which confirms the above argument and consistent with the above calculation: that the reaction zone is significantly larger than flame front thickness. Additionally, from frame 2 to frame 3, the reaction front of the sintering moves ~30 µm in ~55 µs, corresponding to a propagation rate ~50 cm s^−1^ for reactive sintering, a notably faster time than the macroscale flame velocity (~3.3 cm s^−1^) when tracking the flame front during one whole burning event (Supplementary Fig. 2). This observation is further confirmed by the macroscale burn stick experiments (Supplementary Fig. 3) and our recent work^25^, revealing a propagation relationship that is only evident when observed on the microscale (discussed below).

By zooming in, we can actually image a single region that coalesces to a single particle (Fig. 3c). Focusing on an instant in time when the emission is brightest (frame 3 in Fig. 3c) enables us to actually measure both the size of the resulting sintered particle and its temperature: ~30 µm and ~2900 K, respectively. This is consistent with our reaction front thicknesses (~30 µm) discussed above and previously measured temperatures (~3000 K) in macroscale experiments. It is notable that we can achieve a dynamic in-operando temperature measurement of a single sintering particle on the resolution of micrometer. Furthermore, as Fig. 3d shows, there is a ~1000 K temperature difference across the particle in a distance of ~30 µm, which indicates a temperature gradient of 3 ± 1 × 10^7^ K m^−1^, and in the same direction as the flame propagation in this area.

**Fig. 4:**
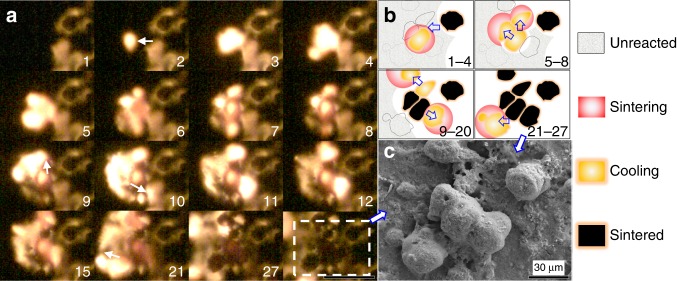
High-speed microscopy images capturing the reactive sintering process in-operando. **a** Series of reactive sintering and ignition snapshots of a group of particles (labeled is frame sequence, 55.55 µs per frame, scale bar: 100 µm) and **b** its corresponding schematic cartoon. Note: The marked arrows are propagating direction. **c** The corresponding scanning electron microscopy (SEM) image of the sintered Al_2_O_3_ particles coated with Cu nanoparticles, as evident by the energy-dispersive spectrometry (EDS) results in Fig. S5

In area B of Fig. 3b, the corresponding particles after sintering were also enlarged and the high-resolution EDS maps are shown in Fig. 3e. From them, we concluded that the main composition of the sintered particle is Al_2_O_3_ (~20 µm) and the coated smaller particles are Cu (in Fig. 3e, <20 nm), which is confirmed by both point-mode EDS and X-ray diffraction patterns (Supplementary Fig. 4). Such small Cu nanoparticles indicate that Cu was vaporized during the reaction due to a flame temperature (≥2900 K) above copper’s boiling point (2835 K). Another 10 particles in Fig. 4b were also examined and the data were listed in Supplementary Table 1. The average sintering time and cooling time of these 11 particles is ~170 and ~265 µs, respectively, consistent with the fact that cooling time is longer than sintering, resulting in a larger cooling zone than sintering zone as evident in Fig. 2. We have tabulated the various length and time scales explored in this study in Table 1.

**Table 1:** Summary of different length and time scales in this study

Length scale of flame propagation	Length scale of sintering	Time scale of sintering	Propagation rate
Flame front thickness: ~30 µm	Sintering zone: ~30 µm	Sintering time: ~170 µs	Spatial propagation rate of reactive sintering: ~50 cm s^−1^
Cooling zone: ~60 µm	Sintered size: ~20 µm	Cooling time: ~265 µs	Burning rate of the macro film: ~3.3 cm s^−1^

As mentioned above and summarized in Table 1, the time scale of reactive sintering is as low as a few microseconds and the spatial propagation rate of reactive sintering is at least a factor of 10 faster (50 vs. 3 cm s^−1^) than the observed macroscale flame velocity. To further explore this difference between micro- and macro-scales, a group of sintering particles were closely monitored as a bridge between reactive sintering and flame propagation. In Fig. 4a, flame propagation is from right to left. An initial burning spot (frame 2) spreads to the surrounding area, where another two sintered particles appear. However, prior to cooling, the adjacent area on the left-top and right-bottom are observed to ignite and then move to the left. The schematic of the above process is demonstrated in Fig. 4b and the final sintered particles are shown in Fig. 4c. More image sequences and the EDS results of final particles can be found in Supplementary Fig. 5. Even though reactive sintering occurs in approximately microseconds, the propagation of the reaction is relatively slow and limiting by the heat conduction and high ignition temperature of Al/CuO (~1000 K)^26^, which in the microscope temporal image show an almost stochastic behavior, despite the overall reaction front moving in a given direction. This suggests that density gradients set up local sintering regions, which comprise heat generation centers. These centers transport energy to neighboring areas that ignite. In this way, propagation is very similar to the general concept of laminar flame theory in which flame velocity ~(thermal diffusivity × reaction rate)^1/2^. The difference being that the macro-scale reaction velocity is limited by the thermal diffusivity between the sintered heat sources.

**Fig. 5:**
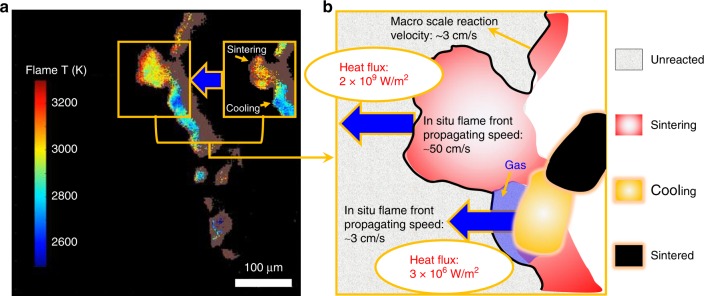
Depiction of the proposed propagation mechanism and the role of reactive sintering. **a** Two typical temperature map snapshots of flame front of Al/CuO nanothermite and **b** its schematic showing heat flux distribution from different stages of reactive sintering particles

## Discussion

To evaluate the above argument, consider that during the passing of the reaction front (1) reactive sintering, (2) cooling, and (3) a final sintered product is observed (Fig. 5a, b). The fact that the flame front consists of these different stages imply an inhomogeneous reaction front with different heat fluxes. We can estimate heat flux ~thermal conductivity × temperature gradient = 10^9^ W m^−2^ in the reactive sintering stage based on the measured temperature gradient and an estimate thermal conductivity for Al/CuO ~60 W m^−1^ K^−1^. This high heat flux supports a rapid front propagating with a velocity as high as ~50 cm s^−1^. However, when reactive sintering has mostly completed (cooling stage starts), the heat flux declines by ~3 orders of magnitude to 10^6^ W m^−2^, owing to much lower thermal conductivity of gas (0.1 W m^−1^ K^−1^), which separates the reacted from unreacted material. Thus, while we see local rapid reaction, these reaction events are slowed down by low conductivity zones, resulting in an over-propagation rate that is considerably lower (Fig. 5b). This calculation indicates that the heat flux in the cooling stage in a reactive sintering process is critical to the macroscale flame velocity.

**Fig. 6:**
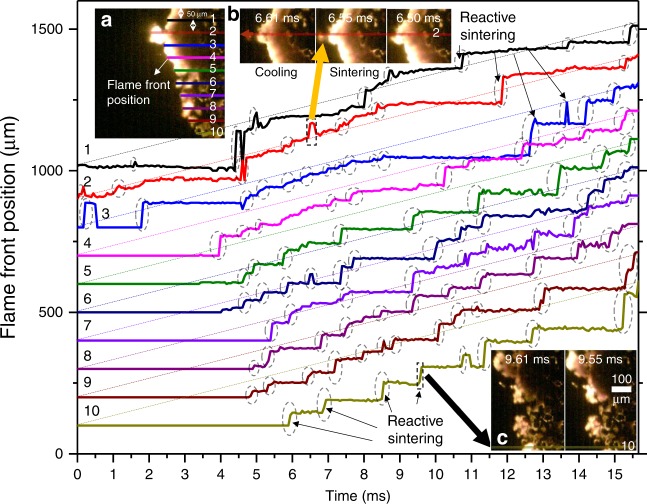
Tracking the time-resolved flame front position (propagation direction is from right to left). Left-top inset (**a**) shows location where flame front is tracked and plotted and ordered 1–10, separated by 50 μm. Inset **b** and **c** show two typical reactive sintering events. The reactive sintering events are annotated with the dashed circles on the line plots

To confirm this, the time-resolved flame front positions were tracked every ~50 μm from top to bottom (10 positions in each frame) and the results are shown in Fig. 6. From this, one can easily distinguish the reactive sintering stage, where the local flame front jumps in a near-step function on the position–time curve. This is consistent with our previous conclusion that reactive sintering occurs much faster than the macroscale flame propagation. After each reactive sintering stage (Fig. 6b, c), a cooling stage (where the flame front position slightly retracts, see inset Fig. 6b) always follows. The different heat fluxes by these two stages drives the flame front propagation at different velocities and, as proposed above, results in a discontinuous flame front. Eventually, the macroscale flame velocity is limited by the cooling stage, which is evident in Fig. 6 since all of the curves have roughly similar slopes.

In this paper, high-speed microscopy/thermometry enables us to observe the in-operando microreaction of Al/CuO nanocomposite thermites. We could visualize and confirm our proposed mechanism for reactive sintering in nanothermite reaction in high spatial (~1 μm) and temporal (~55 μs per frame) resolutions. The temperature map of the flame fronts and the sintering particles were also obtained by a color camera pyrometry of the same images. The flame front thickness was determined from the above results as ~30 µm and the average reactive sintering time and sintered size were obtained as ~170 µs and ~25 µm, respectively. Post-reaction analysis of the exact same area and the corresponding sintered particles were found in SEM, which enables one-to-one composition mapping. For these studies, we can conclude that the local reaction velocity is an order of magnitude higher than macroscale flame velocity. These results imply that local heat generation to create sintered particles has limited heat transport to neighboring areas and is likely the rate limiting step. Heat flux calculations indicate that the macroscale flame velocity was highly dependent on the heat release rate in the cooling stage in a reactive sintering process.

## Methods

### Chemicals and precursor

METHOCEL™ F4M hydroxypropyl methylcellulose (HPMC) was obtained from Dow Chemical Company. Polyvinylidene fluoride (PVDF, average molecular weight: ~534,000) and *N*, *N*-dimethylformamide (DMF, 99.8%) were purchased from Sigma-Aldrich. CuO nanoparticles (~40 nm) were purchased from US Research Nanomaterials. Aluminum nanoparticles (Al NPs, ~85 nm) were purchased from Novacentrix. The active aluminum content is ~81 wt% according to thermogravimetry analysis. All the chemicals were used as received. When preparing a precursor, 125 mg HPMC and 125 mg PVDF were first dissolved in 8 mL DMF and magnetically stirred for ~2 h to get a clear solution. Then, 1761 mg CuO and 489 mg Al NPs were dispersed into the above polymer solution by ultrasonication for ~1 h. The prepared slurry was then magnetically and mechanically stirred for 24 and 1.5 h, respectively, and the precursor was ready to print.

### 3D printing on glass slides

The as-prepared precursor was printed using a Hyrel 30M 3D printer to a commercial cover glass slide (VWR Company, 0.17 mm thickness, 22 mm square) pre-heated to 80 °C. The printing path was an 8 cm × 8 cm domain at ~0.3 mL min^−1^ and writing speed of ~22 cm min^−1^. The details for printing macroscale sticks and corresponding burning characterization can be found elsewhere^25^.

### Additional information about high-speed microscope imaging system

As Fig. 1a shows, the configuration of the high-speed microscope with the high-speed video camera, a ×40 microscope objective (Nikon) with a working distance of 0.66 mm and a high numerical aperture of 0.75. The light collimated by the microscope passes through a beam splitter (ThorLabs) and is focused by the camera lens (Nikon 105 mm Macro) focused at infinity. The third port of the beam splitter cube houses a red LED (630 nm), which is collimated using a plano convex lens at 1ƒ. The collimated beam is reflected by the beam splitter and focused on the sample via the microscope, and the scattered light from the sample is imaged by the camera for focusing purposes. The high-speed video camera (Vision Research VEO710) recorded at framerates of 18,000 frames s^−1^, with an exposure of ~55 µs.

### Color video pyrometry

Color ratio pyrometry was performed to estimate temperature of the burning films^21^. By taking ratios of raw color channel intensities, as imaged by a red/green/blue Bayer filter and CMOS assembly, dependency on most variables associated with light intensity (e.g., solid body angle) are eliminated, except for those pertaining to channel gain, emissivity, and spectral response of the optical components (microscope lens, camera lens, beam splitter, Bayer filter, CMOS sensor) at individual wavelengths. The camera was calibrated for temperatures ranging from 773 to 4773 K with a Newport Oriel 67000 Series Blackbody Infrared Light Source using Planck’s Law and the associated graybody assumption. MATLAB was used to extract raw pixel values, de-mosaic the Bayer filter to recover values for RGB at each pixel, and calculate temperatures based on color intensity ratios of the pixels. Three color ratios (green/red, blue/green, and blue/red) were simultaneously used to estimate temperature by minimizing their summed error from theoretical ratios with a nominal error <~110 K. For the figures that show temperature of a single sample as a function of time, only unsaturated pixels above the black level and within the error threshold are used to report mean/median temperature of the frame for a contiguous area of at least 10 acceptable pixels.

### Morphology characterizations

The microstructure of the printed samples was investigated using a Hitachi SU-70 SEM coupled to an EDS. The printed samples were sectioned in liquid nitrogen and attached to a carbon film on an SEM stage. The SEM and EDS mapping images of 90 wt% Al/CuO nanothermites were shown in Supplementary Fig. 1.

## Supplementary information

Supplementary Information

## Data Availability

All the data that support the findings of this study are available from the corresponding author upon reasonable request. The MATLAB code used in this study for color video pyrometry is available from the corresponding author upon reasonable request with research purpose.
