# Supplementary material for: In-operando high-speed microscopy and thermometry of reaction propagation and sintering in a nanocomposite
Source: Nat Commun. 2019 Jul 10;10:3032. doi: 10.1038/s41467-019-10843-4 (PMC6620330; doi:10.1038/s41467-019-10843-4)
Supplement: Supplementary file 1 — Supplementary Information [file 41467_2019_10843_MOESM1_ESM.pdf]

In-operando high-speed microscopy and thermometry of reaction propagation and sintering in a nanocomposite, Wang et al.

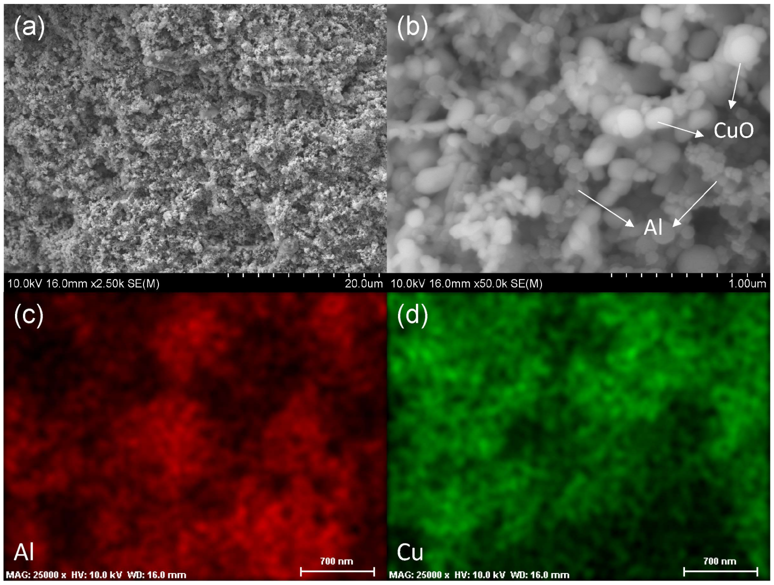

Supplementary Figure 1 Low (a) and high (b) SEM images, EDS results (c, Al; d, Cu) of a cross-sectional Al/CuO/HPMC/PVDF film.

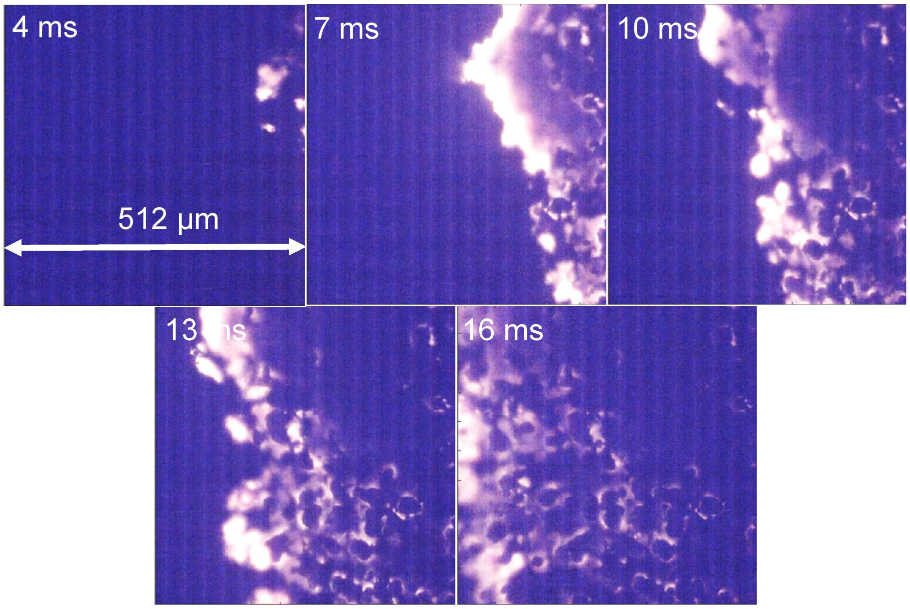

Supplementary Figure 2 The flame propagation observed in a 512 μm×512 μm zone.

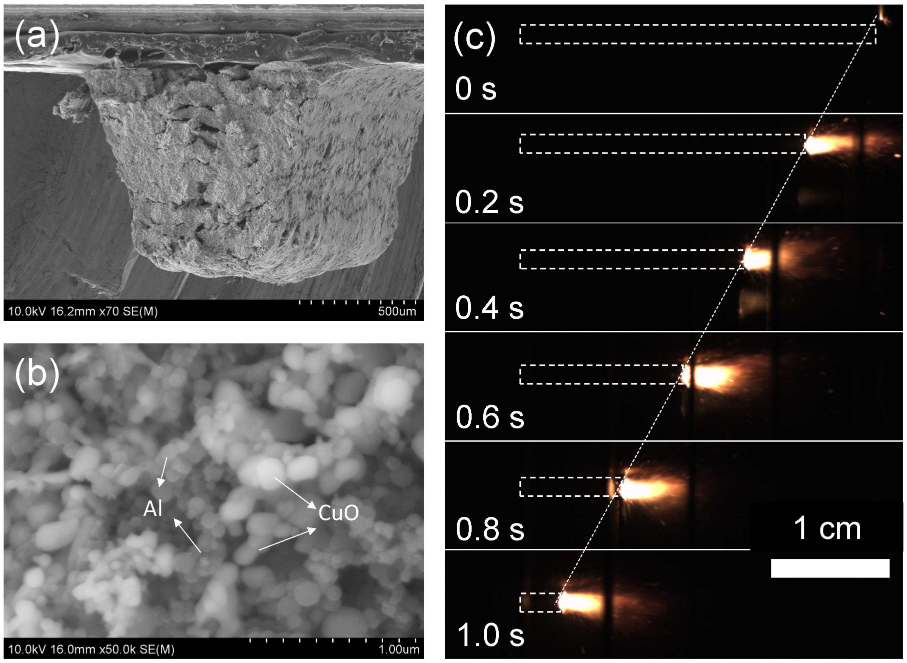

Supplementary Figure 3 Low (a) and high (b) resolution SEM images of the printed Al/CuO (90 wt.%) stick, and its burning snapshots in argon atmosphere (1 atm).

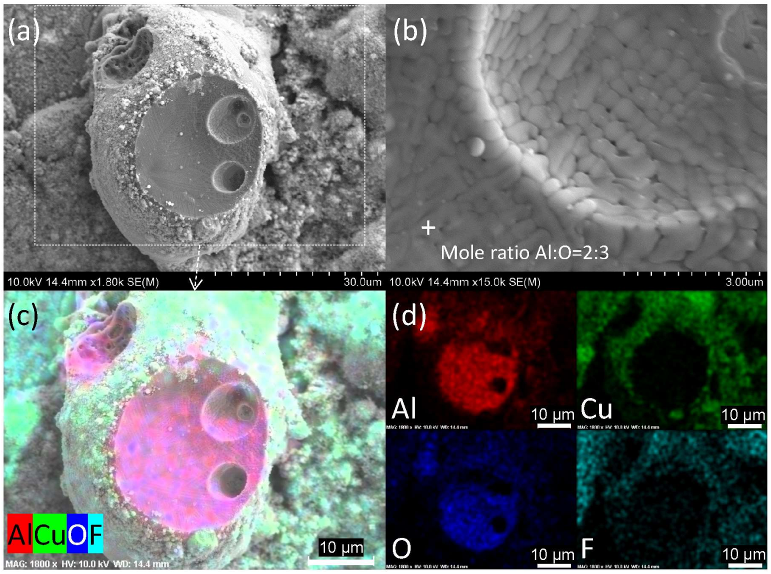

Supplementary Figure 4 Low (a) and high (b) SEM images, EDS results (c and d) of a cross-sectional sintered particle. Based on a simplified estimation, one ~25-micron sintered Al_2O_3, was formed by ~30 million ~80 nm Al NPs. The fine Al_2O_3 inside (~300 nm) was sintered by ~50 Al NPs.

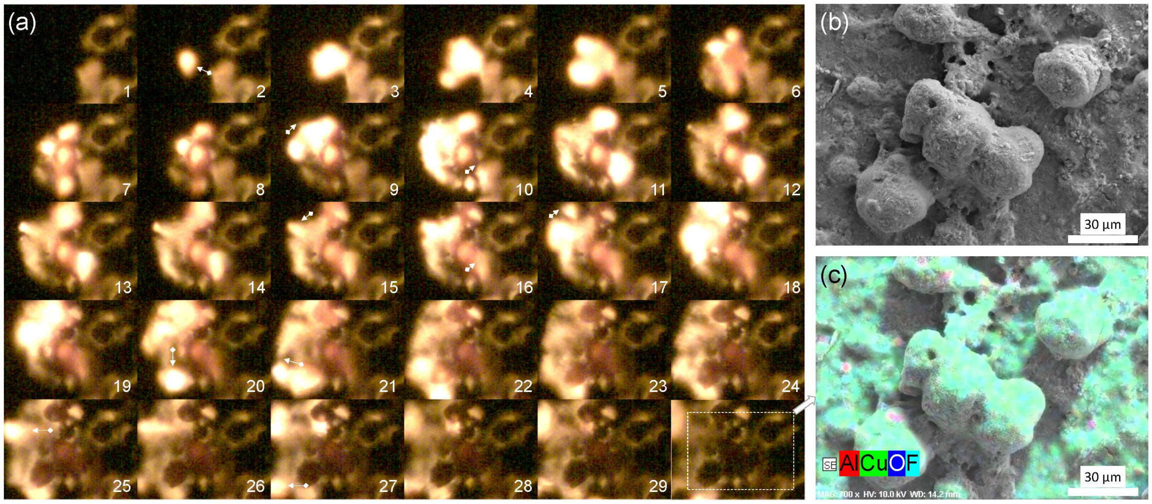

Supplementary Figure 5 Series of reactive sintering and ignition snapshots of a group particles (a, labeled is frame sequence, 55.55 µs per frame). Note: the marked arrows are propagating direction. The corresponding SEM image (b) of the sintered Al_2O_3 particles coated with Cu nanoparticles, as evident by the EDS results (c).

Supplementary Table 1 Sintering time, cooling time and size distribution of typical particles. Note: The cooling time for particle 1#, 2# and 4# is invalid because they were reheated by surrounding sintering particles.

Particles #	Sintering time (us)	Cooling time (us)	Size (um)
1	170	1780 (invalid)	35
2	170	1220 (invalid)	20
3	110	110	35
4	220	1060 (invalid)	30
5	220	280	20
6	170	390	25
7	110	170	15
8	220	440	35
9	170	170	20
10	220	280	25
11	110	280	20
Mean	170	265	25
Error	50	110	7
